# Providing health care in politically charged contexts: a qualitative study about experiences during a public collective hunger strike of asylum seekers in Germany

**DOI:** 10.1080/17482631.2021.2018770

**Published:** 2021-12-31

**Authors:** Dominik Haselwarter, Verina Wild, Katja Kuehlmeyer

**Affiliations:** a Institute of Ethics, History and Theory of Medicine, LMU Munich, Munich, Germany; b Ethics of Medicine, Medical Faculty, University of Augsburg, Augsburg, Germany

**Keywords:** Physician role, role conflict, doctor-patient relationship, decision-making, hunger strike, asylum seekers, dual loyalty

## Abstract

**Purpose:**

Role expectations of physicians providing health care for hunger strikers have been discussed in the context of prisons and detention centres. Ethical guidance for physicians in these situations is codified in the Declaration of Malta. In the last years, new forms of *collective, public* hunger strikes of asylum seekers have occurred. We have aimed at reconstructing the experiences of health-care personnel involved in one of such cases.

**Methods:**

Semi-structured interviews with nine participants (physicians and paramedics) that had been involved in a public collective hunger strike of asylum seekers in Germany were conducted.

**Results:**

We identified three health-care provider groups: voluntary physicians, emergency service providers and medical consultants for the authorities. Role conflicts arising from multiple loyalty situations with obligations towards different stakeholders (e.g., strikers, employers, authorities) were perceived as the greatest challenge especially for voluntary doctor and emergency service provider participants. Such conflicts culminated in feeling instrumentalized for political goals.

**Conclusion:**

The results illustrate that professional challenges in the health care during a public collective hunger strike differ in various aspects from those described in the literature on custodial settings. We recommend expanding and adapting the medico-ethical guidance.

## 1. Introduction

A hunger strike is by definition “food refusal as a form of protest or demand” (World Medical Association [WMA], 2006, p. 36). Hunger strikes evolve in complex situations and can pose serious health risks to protesters. Practical and ethical guidance has been developed for doctors, such as the Declaration of Malta by the World Medical Association (WMA, 2006, 2017). Its basis is a critical reflection of the physician’s role, the doctor-striker relationship and processes of decision-making. It claims that hunger strikes are “particularly undertaken by people in custodial settings who lack alternative means to gain attention” (WMA, 2006, p. 36), and accordingly it focuses on hunger strikes in detention. An extensive narrative review including reports from twelve countries (Gulati et al., 2018) almost exclusively focuses on hunger strikes in detention—either in prisons and jails or in immigration detention centres.

The few existing reports about cases of hunger strikes in non-custodial settings rarely report results of empirical studies (e.g., Dixon, 1999; Kenyon, 1999; Uprety et al., 2016). In particular, research with regard to cases of collective hunger strikes taking place in public is lacking. Such a situation differs substantially from a situation of hunger strikers in detention: strikers can leave the situation at any time, they can communicate with others at their own request, and they have greater degrees of freedom to choose a doctor they trust.

In order to expand the background knowledge that ultimately forms the basis of policy-making, we provide insights into a case of a collective hunger strike of asylum seekers on a German public square. Our paper aims at describing the experiences of health care professionals, to learn about their perspectives and challenges regarding the delivery of health care. We were especially interested in perceptions of their professional role, potential role conflicts and their relationships with the hunger strikers and others on the site.

The results of our research allow for an identification of context-relevant challenges. Such a lay of land study can help to define the status quo in a field of care and identify challenges that need further attention (Kon, 2009). Specifically, our study can help identify potential ethical challenges and dilemmas. This may be the basis for developing policy documents and ethical guidelines that address professional-ethical challenges in health care provision in politically contested areas such as in the case of public collective hunger strikes. Furthermore, our publication can contribute to a better support, training and preparation of health care professionals involved in such events.

## 2. Background

### 2.1 Health risks of different forms of hunger striking

Hunger strikes involve serious risks to the health of strikers that demand medical attention. Three types of hunger strikes can be distinguished: *partial fasting*, where the hunger striker abstains from solid food but takes fluids, sugar and vitamins; *total fasting*, in which fluids are ingested, but no solid food; and *dry fasting*, in which strikers ingest neither food nor fluids (Vanobberghen et al., 2019). All three forms can cause serious damage to health, both acute and long-term. While dry fasting leads to death by dehydration within approximately one week (WMA, 2006), the average survival time for a normal-weight person practicing total fasting is 2–3 months (Chalela & Lopez, 2013). Severe neurological and psychological changes can be induced by fasting, such as alterations of consciousness, amnesia, psychotic episodes or delusions (Başoğlu et al., 2006; Fessler, 2003; Gétaz et al., 2012), which can severely impair one’s decision-making capacity (Fessler, 2003). The few studies on long-term health effects of hunger strikes mention renal failure, starvation colitis, Wernicke-Korsakoff syndrome and post-traumatic stress disorder (Başoğlu et al., 2006; Chalela & Lopez, 2013). A feared complication after resuming the intake of nutrition is the refeeding syndrome, a life-threatening metabolic disturbance that occurs as a result of reinstitution of nutrition after a long period of fasting (Eichelberger et al., 2014). This calls for medical attention even after the end of the strike, in order to prevent additional harm to the striker.

### 2.2 Doctors’ professional role expectations during health care for hunger strikers

While doctors’ professional role expectations are well-defined in the regular health-care setting, they appear rather ambiguous in the unusual situation of a hunger strike. Beside confidentiality and practicing *lege artis*, patients can expect from their doctors that they will promote their well-being. However, in a hunger strike, the perspectives of what promoting short- and long-term well-being entails, may differ. The strikers are risking their health, and even their lives, in order to raise awareness and achieve a certain goal (Crosby et al., 2007; Karni, 2015; Reyes, 2007) which they consider to be in their best interests. Strikers may even prioritize a collective political goal over their own health. This stands in contrast to general expectations, i.e., that a person with health problems seeks the help of an expert to ascribe (or not) a disease to him/her that allows “a patient” to take on “the sick role” (Parsons, 1951) and that a patient’s cooperation is directed towards a quick recovery from illness to resume his or her role in society.

A requirement for the credibility of that judgement is that it is based on an informed choice e.g., about a termination of the strike or an application of medical measures. To enable that, freedom from coercion, a sharing of information and an exchange of values between the doctor and the striker and the autonomous decision of a striker are key (Crosby et al., 2007; Gulati et al., 2018; Jacobs, 2012; Kenny et al., 2004; Oguz & Miles, 2005; Reyes, 2007; Silove et al., 1996; WMA, 2017). Another important requirement for the strikers’ decisions to be autonomous is that strikers are competent to make such decisions. Doctors have to assess whether strikers gauge the exact scope of the decision and make sure that the strikers’ competence is not significantly impaired by severe mental illness or other pathological mental states (García-Guerrero, 2013; Gulati et al., 2018; Kenny et al., 2004; Silove et al., 1996; Wei & Brendel, 2010; WMA, 2017). Confidential conversations with the hunger strikers are required for this assessment, where it is within the physicians’ responsibility to assess the strikers’ true intention and will, independently of the influence of others (especially in decisions about the rejection or acceptance of possible medical interventions). In such conversations, it is also necessary to provide information on possible consequences of hunger striking to the health and well-being to the strikers. Physicians have to respect the strikers’ right to well-considered rejection of potentially life-saving medical interventions which is referred to as “informed refusal” in the context of hunger strikes (Gétaz et al., 2012; Gulati et al., 2018; Oguz & Miles, 2005). At the same time, acceptance of the informed refusal of an essential medical measure is in conflict with the ethical duty of beneficence. Furthermore, physicians should have sufficient opportunities to examine and evaluate the hunger strikers’ health status prior to hunger striking to be able to foresee and address personal additional risks involved in the action. In light of these responsibilities, a good doctor-striker relationship and successful communication built on mutual trust are crucial (Hoff & Collinson, 2017; Irmak, 2015; Reyes et al., 2013). Trust is particularly critical in relationships characterized by a lack of choice for the patient or in a context of asymmetry, a situation that applies to the context of hunger strikes (Filc et al., 2014). Trust can be understood as a feeling of reassurance and confidence towards the physician and it implies the patients’ expectations that doctors will do their best for their patients (Rasiah et al., 2020). To build and maintain trust, doctors will have to demonstrate to patients that they are recognizing their vulnerabilities and that they are acting in their best interest.

To provide health care also requires physicians to preserve their clinical independence and to direct their actions mainly towards their individual patients’ interests rather than towards the interests of the physicians’ employers or the authorities. The various demands on a doctor in a hunger strike may lead to role conflicts (Kenny et al., 2004). The problem of maintaining clinical independence in a setting of conflicting loyalties is described as a key challenge in health care for hunger strikers in custodial settings (Briskman & Zion, 2014; Gulati et al., 2018; Sanggaran et al., 2016). Dual loyalty situations can occur when health care professionals are confronted with duties towards the hunger strikers as their patients and expectations of the employing authorities. As mentioned above, doctors are ethically obliged to act in their patients’ best interests and to respect their autonomous decisions even with regard to the rejection of medical treatment. Authorities involved in the strike might expect health professionals to save the strikers’ lives at all costs, which could entail life-prolonging interventions such as the administration of artificial nutrition and hydration or cardiopulmonary resuscitation against the will of a hunger striker. These tensions are perceived as a major source of role conflicts (Gulati et al., 2018). While some ethical guidance exists by the WMA (such as weighing the respect for the autonomy of a competent striker vs. beneficence), the situation of a public collective hunger strike is so far neither well-understood nor explicitly addressed by policy.

### 2.3 Public collective hunger strikes of asylum seekers in Germany

In various German cities, organized groups of asylum seekers have gone on hunger strike in order to emphasize their protest and put pressure on the authorities. Since 2012 at least 15 of such hunger strikes have been reported (e.g., Amjahid & Kather, Glas, 2017; Glöde & Böhlo, 2015; Gschwendtner et al., 2016; Kastner, 2014; Odugbesan & Schwiertz, 2018; Ruedin et al., 2018). They were carried out by a few individuals or large groups of up to 150 asylum seekers (Glas, 2017), some of them lasting up to 17 days (Glöde & Böhlo, 2015).

Hunger strike can be understood as “a form of protest by people who lack other ways of making their demands known” (WMA, 2017, p. 1), as genuinely political acts and as a “political tool [which is] part of political struggle” (Filc et al., 2014, p. 231). The general demands of striking asylum seekers in Germany were, amongst others, the recognition of asylum, protection against deportations, the right to work and study and the improvement of living and housing conditions (Glöde & Böhlo, 2015; Odugbesan & Schwiertz, 2018; Ruedin et al., 2018). The demands are understandable in light of the restrictive German asylum policy. Human rights and welfare organizations are regularly criticizing German asylum policy, e.g., for inhuman housing conditions, isolation from the general population, limited access to education and legal and social counselling (Pichl, 2017). In 2019, about 143,000 people filed an application for asylum in Germany, while the acceptance rate was 38% for the same year (Bundesamt für Migration und Flüchtlinge, 2020).

Public collective hunger strikes of asylum seekers, although not centrally coordinated across the country, often followed a similar pattern. Organized groups of asylum seekers began hunger strikes on public, highly frequented places in German cities or in front of the buildings of public authorities or political institutions. In most cases, the strikers set up camps and barriers to distinguish themselves from outsiders, where they expressed their demands on banners, similar to political demonstrations. Sometimes the hunger strikers were supported logistically and politically by political activists (Glöde & Böhlo, 2015).

During public and collective hunger strikes health care was often provided by various groups of medical professionals. In addition to regular medical forces such as emergency physicians and ambulance services, as well as regular inpatient care in the event of hospitalization, health care was often additionally provided by voluntary health professionals that were not employed by any institution but affiliated with professional associations. In some cases, they also politically engagedin the goals of the asylum seekers (Verein demokratischer Ärztinnen und Ärzte, 2014). Similar to demonstrations or political rallies, public collective hunger strikes were considered political gatherings by the authorities and were hence toleratedon the basis of the right to freedom of assembly. Nevertheless, there were numerous cases of forced evictions of hunger strikers by order of the authorities (Glöde & Böhlo, 2015; Gschwendtner et al., 2016; Kastner, 2014).

Although there is only little empirical research on health care in public hunger strikes so far, the setting seems to differ significantly from custodial settings in various aspects. A hunger strike in the public sphere is less easy to control from the outside, in contrast to a hunger strike in a custodial setting. The strikers and the surrounding groups are more visible and hence more exposed to the general public and the media, which can put additional pressure on the negotiations with the authorities. As long as authorities tolerate the protest, the strikers are in control of the setting. They are in a position to decide, whether they accept medical personnel within the camp or not, whether they create spaces for confidential conversations or not and whether they want to accept outsiders in or send insiders out. A collective protest is also possible in the custodial setting, but a collective public protest is more complex, involving more parties in a dynamic way, including supporters and voluntary helpers, giving more opportunities for group dynamics and greater risks for group pressure. Especially the presence of voluntary health professionals appears to represent a significant difference from hunger strikes in detention, where physicians either aredirectly subordinate to prison authorities, or at least their access to the hunger strikers is controlled by the authorities (Vanobberghen et al., 2019, 2020). We chose to study one case of a public and collective hunger strike of asylum seekers that we consider to be a precedent for similar events.

### 2.4 The case in the focus of our research

Among the public collective hunger strikes that took place in Germanyin the last decade, we chose case of a hunger strike that can be regarded as outstanding in several aspects. It was one of the first cases where a large group of asylum seekers went on hunger strike and presented unprecedented challenges to the responsible authorities and the health professionals involved. Due to the dramatic nature and the increasing escalation of the events the resonance in the media and on a political level was enormous.

The hunger strike we investigated took place at a public square in a German city. A group of about 50 asylum seekers made various demands on the authorities; first and foremost was the recognition of asylum for all participants. The hunger strikers were accompanied by a group of supporters who declared solidarity with their goals and were permanently present at the hunger strike without participating in the fasting. The hunger strikers and their supporters erected a small camp of tents to protect themselves (e.g., against bystanders, media reporting and weather). Access to the camp was controlled by the hunger strikers and their supporters. The city administration convened a crisis committee consisting of representatives from politics, police, administrative authorities and members of public health services. In the absence of an agreement, the strikers declared to go on a dry hunger strike (dry fasting) on the fourth day. As no agreement was reached between the conflicting parties, the camp was forcibly evicted on the eighth day of the strike. As a justification for the forced eviction the public authorities stated that the life and health of the strikers could no longer be guaranteed due to the continued dry fasting. The forced eviction took place without prior announcement and was executed by a large contingent of police and with the help of public emergency services, including coercive measures against the hunger strikers and their supporters. The hunger strikers were transported to hospitals. None of them died.

As stated above, we are now aiming at to describe the experiences of the health-care professionals involved in the provision of health care during that hunger strike in order to learn about their perspectives on the delivery of health care.

## 3. Methods

### 3.1 Design

We chose a qualitative research design because it can be used to describe phenomena “from within”, with a focus on the subjective perspectives of the acting subjects (Flick et al., 2008). Its flexibility in adjustment to the study object allows the exploration of phenomena characterized by diversity, plurality and mutability. Since the dynamics of the hunger strike did not allow us to conduct an observational study, we relied on retrospective interviews. This description follows the consolidated criteria for reporting qualitative research (COREQ-) checklist (Tong et al., 2007).

### 3.2 Methodology

We used interviews as a structured form of conversation to explore the participants’ experiences, interpretations, attitudes and opinions and applied qualitative content analysis (QCA, Mayring, 2015). We understand QCA as a qualitative research method based on social constructivism rather than on positivism (Stamann et al., 2016). QCA, described as “qualitative oriented category-based text analysis”, is a method particularly suitable for the analysis of interview protocols (Stamann et al., 2016). It aims to capture not only the “manifest meaning” but also the “latent meaning” of conversations and permits inductive-deductive analysis (Mayring, 2015).

### 3.3 Sampling and recruitment

Participants were selected by purposeful sampling, aiming to select individuals or groups that have relevant knowledge and experience (Palinkas et al., 2015). Accordingly, we approached health-care professionals involved in one case of a public collective hunger strike in Germany. We deliberately refrained from detailing the participants’ situation and recruitment in order to protect their anonymity. We aimed at representing the full spectrum of professional roles involved by approaching a) physicians and paramedics and b) officially appointed and voluntary medical personnel. The first participants were contacted directly. Participants then acted as multipliers (snowball sampling). Of eleven eligible persons we included nine, two declined. DH arranged and conducted the interviews. The participants did not have prior relationships with DH.

### 3.4 Data collection

In face-to-face interviews participants were asked to retrospectively describe their experiences with the health-care provision for hunger strikers. We used a semi-structured interview grid, following Cornelia Helfferich (2011). The content of the questions was based on literature on the medical care for hunger strikers (Crosby et al., 2007; Dougherty et al., 2013; Fessler, 2003; García-Guerrero, 2013; Gétaz et al., 2012; Gregory, 2005; Gross, 2013; Gulati et al., 2018; Jacobs, 2012; Reyes, 2007; Silove et al., 1996; Wei & Brendel, 2010; WMA, 2006, 2017), and on literature and media coverage on asylum seekers’ hunger strikes in Germany. All authors revised and discussed it. In the interviews six key themes were explored: (1) the participants‘ reasons or motivation to be present at the hunger strike, (2) their perceived and actual tasks, (3) their interaction with the strikers, (4) their interaction with other actors, (5) the comparison of the hunger strike situation with their “regular” medical practice and (6) an overall assessment of the situation.

The interviews were conducted three to four years after the hunger strike. They took place in a quiet atmosphere in different locations chosen by the interviewees: participants’ offices at their workplaces (n = 5), a university building (n = 2), the participants’ home (n = 1) and a café (n = 1). All participants were interviewed alone, except in the interview of one physician where on his/her request a nurse was also present in the room. The interviewer (DH) holds a Bachelor’s Degree in Psychology and was in his fourth and fifth year of medical school during the phase of data collection. He was trained in conducting qualitative interviews by KK (psychologist/bioethicist) and VW (physician/bioethicist) who were experienced in conducting qualitative interviews and in training novice researchers. The interviews were digitally recorded and transcribed verbatim following simple transcription rules (Dresing & Pehl, 2013). In order to ensure anonymity, audio files were deleted after transcription and anonymized, altering names, places and other potentially recognizable information.

### 3.5 Data analysis

Data analysis was conducted according to Mayring (2015) with the help of a specially developed coding grid and with the software *MAXQDA*. The interview material was worked through until no further categories were added to the category system. Then it was discussed and adjusted, resulting in five main categories: [1] assignment description and role perception, [2] medical tasks and challenges, [3] interaction with the hunger strikers, [4] interaction with other actors, [5] role-conflicts. The entire interview material was coded. Selected quotes were translated from German into English and proofread by a translator. For readability, we edited grammatical irregularities, colloquial languages and neologisms. Truncation of quotations and explanatory comments inserted by the authors are indicated using brackets […]. A triangulation of perspectives was used to enhance the quality of the interpretations.

### 3.6 Research ethics

The participants were informed orally and in writing about the study’s aim and procedure, the voluntary nature of the participation, their right to withdraw from the study, the intention to record the conversation, confidentiality, anonymization of the material and data protection. Written informed consent was obtained from all participants. The study received ethics approval from the Research Institutional Review Board at the Medical Faculty of LMU Munich (312–16). One central ethical challenge is to ensure the participants’ anonymity, as the interviews are touching sensitive issues with regard to the participants’ engagement in the case which, in some cases is the subject of ongoing conflicts. Since hunger strikes are rare events, we will describe certain aspects of the situation and the participants rather vaguely or will distort attributes.

## 4. Results

### 4.1 Participants

The sample consisted of nine health-care professionals. Through the participants’ self-identification with certain collectives, we identified three different groups of participants who built different relationships with the strikers: 1) Voluntary health-care professionals who were on site without being sent, employed or paid by any institution and who were directly involved in the provision of health care to the strikers. 2) Members of the public emergency services who were sent through a centralized management of those services and were on site as part of their professional activities being paid by their employing institution. They were also directly involved in the provision of health care to the strikers 3) Medical consultants who were employed and paid by the authorities and who were not actively participating in medical care, but assessing and monitoring the health status of hunger strikers and passing on information to the relevant authorities. Table I provides an overview of the three groups.

**Table I.** Description of the sample

Group	Number	Profession	Professional experience (range)
Voluntary physicians	3	3 physicians	< 3 years to > 30 years
Public emergency services	4	2 physicians 2 paramedics	10 years to > 30 years
Medical consultants for the authorities	2	2 physicians	> 10 years

### 4.2 Perspectives of the involved health personnel

Here we show the three perspectives of the three medical groups in more detail.

#### 4.2.1 Voluntary physicians

[1] Assignment description and role perception

Three physicians offered their voluntary service after having heard through media coverage or personal contacts that the group of hunger strikers were looking for physicians. Two of them reported that they had always been interested in refugee and migration related issues. The other reported his/her involvement as follows:

Participant (3): *“[A]n acquaintance called me […]: ‘We’ve got a pregnant woman here and they declared some kind of hunger strike and we’re a little bit worried now—could you just come by?’ […] I […] checked her, found out that she was doing well so far […] I came out of this tent and there was a line of fifteen people. […] [I] came home in the evening and I remember saying to my [partner] then: ‘I have to go back tomorrow because there are people who need me.’”*

Participants of this group reported a feeling of responsibility for the hunger strikers’ health and well-being.

Participant (3): “*We weren’t here as supporters, we weren’t here as opponents, we were here to make sure that no people got hurt, no matter who.”*

While he/she regarded him-/herself as “neutral” and without own political goals, he/she reported that the engagement of some of his/her colleagues was politically motivated with the aim of supporting the political goals of the strikers. Initially, none of them was assigned or paid by an employing institution.

[2] Medical tasks and challenges

The voluntary doctors described themselves as the first contact persons for the strikers’ health problems. They were a small self-organized group of health professionals (doctors, nurses or paramedics) who were meeting daily. They made a shift plan ensuring their service around the clock. Their priority seemed the provision of first-tier medical care, such as regular checks of vigilance and vital signs during daytime and night-time and taking care of smaller medical problems. In case of more severe issues, such as when a hunger striker showed a significant reduction of vigilance or collapsed, they called in the public emergency services. The participants of this group reported that they had no experience with medical care for hunger strikers or any comparable situation. They perceived their own activity partly as improvised and uncoordinated. Two reported that they had to work under poor conditions with scarce resources. They reported psychological and physical strain, as the number of patients in need of treatment increased.

[3] Interaction with the hunger strikers

Participants reported having unhindered access to the camp and partly also trustful relationships with the hunger strikers. In their experience, medical measures and hospital admissions were accepted by the hunger strikers:

Participant (1): *“If someone was in pain, if someone was feeling bad and they were asked: Do you want to go to the hospital? […] they agreed. There wasn’t anyone, so I can’t remember anyone saying: ‘No, I don’t want that now, I don’t want anything’.”*

One participant stated that one of his/her personal conditions to maintain his/her involvement in the medical care was that his/her medical recommendations—such as hospital admissions—were accepted by the hunger strikers.

They criticized having had only little opportunity for confidential conversations with the strikers. Conversations would have been regarded as useful for anamnesis, obtaining information about pre-existing illnesses, medical history and medical treatment:

Participant (1): “*But actually, I think in such a situation a conversation would be so important that one could calmly clarify with the person: ‘Hey, what’s going on? Do you want that? Would you like that? Do you know what the consequences are?’”*

Participants of this group reported that they were not sure if all of the hunger strikers had fully understood the medical implications of dry fasting, including possible long-term-damage to their health.

Furthermore, they had the impression that group dynamics and peer pressure might have played a role in the individual’s decision to maintain hunger striking, as the influence of leaders on others could not be excluded.

Participant (3): *“I saw people who were pushed to do things that they certainly would not have done on their own. And that was something that really concerned me, because it’s a high price to pay. I mean, in the end, it’s all about human lives.”*

[4] Role conflicts

Two of the voluntary doctors expressed discomfort and doubts about their own role with regard to how they would personally cope if hunger strikers were to be seriously harmed or even died:

Participant (3): *“That’s what I was asking myself all week long: Are there people dying now and how many? And how will you deal with that?”*

One participant described a conflict between the strikers’ individual and collective interests:

Participant (3): “*I had one case that I remember well, where someone in the tent actually asked me if I could get him/her some water. And that was one of the most difficult moments for me as I really found myself in a big conflict. I mean, on the one hand, medically: sure, someone is asking for help and tells me what he/she needs. But at the same time, I am also aware of what the rules of the game of this camp are and how everything is working here. Finally, I decided to act according to his/her wish because I primarily felt committed towards HIM/HER. Somebody else noticed that and there was a big scandal. And after that I had to commit myself that I will not give anyone liquid anymore, otherwise [I] won’t get into the camp anymore.”*

The participant described that he/she had to promise to the assembly of all hunger strikers not to give drinking water to the strikers anymore, even if this was demanded by individuals. He/she accepted this demand—though with great discomfort—in order to maintain access to the camp and provide health care to the hunger strikers.

Another role conflict was described in relation to a request for measures to alleviate symptoms caused by dry fasting:

Participant (3): *“Well, I’m not giving them painkillers or anything. Because this was always such a discussion: ‘I have a headache!’—‘Yes, of course, my friend, you haven’t had a drink in two days, you’re on a hunger strike. I’m not going to give you drugs and dope you so you can go on hunger strike longer.’ This isn’t the assignment here, not as I understand it. But that’s the balancing act.”*

The partiality of physicians was an important issue in their relationship with the strikers. One participant described that the strikers’ perception of his/her role changed on the fourth day when he/she received payment by the authorities for his/her engagement in the health care for the hunger strikers. He/she argued that by cooperating with the authorities, he/she gained access to medical equipment provided by the authorities. When this became known by the hunger strikers, he/she was no longer seen as a person of trust and was no longer permitted access to the camp during the last days of the hunger strike.

#### 4.2.2 Public emergency services (doctors and paramedics)

[1] Assignment description and role perception

Participants of the public emergency services group were involved due to their professional assignment by their employing organization. They were located outside of the camp and available at any time. They described their role as being responsible for offering medical care and for transport to hospitals. Subjectively they saw it as their duty to provide emergency care to the hunger strikers to prevent harm.

[2] Medical tasks and challenges

The participants reported providing health care for the hunger strikers jointly with the voluntary physicians. The public emergency services seemingly were called into the inner circle of the camp in case of serious medical problems. In such situations, they reported being confronted with clinical symptoms caused by dry fasting such as dehydration and metabolic disbalance, reduction of vigilance or collapse. An intervention was performed if necessary, e.g., administration of liquids or transport to a hospital. Participants reported that most strikers resumed the hunger strike after being treated in hospitals. As the hunger strike continued, more seemed to be in critical conditions. This was described as generally easy to handle for health-care personnel experienced in emergency medicine. When more strikers collapsed, the participants experienced difficulties in keeping the situation under control. All noted that this was an unprecedented situation.

[3] Interaction with the hunger strikers

One emergency doctor stated that he/she had unhindered access to the camp, the others stated that they were only allowed access to the hunger strikers in case of a medical emergency. These participants reported that their proposed interventions were always accepted by the hunger strikers. One made that clear:

Participant (4): *“[…] no one there wanted to die. That they wanted the maximum and optimal therapy, at least I cannot remember that anything else was ever requested “.*

Several participants reported that it was not always possible to assess the hunger strikers’ current health status beyond the acute symptoms. Yet, language barriers were not perceived as a major obstacle, as interpreters were available.

[4] Role conflicts

Several participants ascribed negative feelings to a role conflict between obligations towards the hunger strikers and the requirements by their employers or the public authorities. As one of the emergency doctors puts it:

Participant (4): *“I mean, it was generally a difficult situation as, on the one hand, I was on the municipality’s payroll and, on the other hand, the hunger strikers trusted me to some extent.“*

This conflict culminated when they had to participate in the forced eviction of the camp. The decision to terminate the hunger strike by coercive eviction was made by a crisis committee installed by the authorities. At that time, the dry hunger strike had gone on for several days and more hunger strikers were hospitalized. The eviction was executed by a large number of police forces with the support of the public emergency services who were supposed to carry away the physically weakened, vulnerable strikers.

The public emergency services were involved in the forced eviction to avarying extent, but all participants of this group perceived it as a major point of conflict. One physician was ordered by his/her employing public institution to triage the hunger strikers during the forced eviction in order to organize hospital admissions. He/she expressed a strong feeling of discomfort when “*getting caught between the fronts*”—being trusted by the hunger strikers on the one hand, and the expectations of the employer on the other.

Paramedics expressed similar perceptions with regard to their role in the forced eviction. As part of the official medical personnel they were requested by their employer to participate by carrying the hunger strikers to ambulance cars and transporting them to hospitals against their expressed will. This was perceived as a breach of their professional role:

Participant (6): *“I’m not forcibly pulling anyone out. I am not a policeman. […] Coercive measures are taken by the police.”*

Several participants of this group mentioned—independently from each other and without being asked about it—the term “instrumentalization” in order to describe their experiences during the forced eviction. They described being instrumentalized by the public authorities for their political goals. One of the emergency doctors also used the term “instrumentalization” when speaking about his/her role in the municipality’s crisis committee:

Participant (4): *“I was then also appointed to these committees […] And then luckily there was also [a person] my actual boss […] and [he/she] always said to me: ‘Be careful, they are instrumentalizing you! Don’t make a statement!’”*

Interviewer: “*Who is instrumentalizing you?”*

Participant (4): *“The municipality. Just don’t say anything. They should do it themselves, I should hold back. […] And I did hold back, I’m not crazy. So, I mean in the end then it is: “He said … ” And then there are 300 policemen. And I don’t want anything to do with it. […] And, of course, the city and the police want someone who says: “This is life-threatening.” Which it certainly was. But that is something everyone knows. That’s where they want a medical statement. And in the course of time the pressure became stronger and stronger. So that one says: okay, one tries to tickle out a statement that leads to 300 policemen evicting the camp“.*

Some shared the impression that the authorities tried to end the hunger strike in order to prevent deaths and any associated media coverage. The suspicion was expressed by one participant that the medical situation had been dramatized for the media and that reasons had been specifically sought to legitimize forced eviction.

Some of the participants of this group also expressed the feeling of being instrumentalized for the political goals of the hunger strikers and their supporters. One paramedic stated that the hunger strikers were relying on the “backing” by the medical personnel as a part of their political strategy.

Participants of this group expressed a strong feeling of discomfort with their role in the hunger strike. As one paramedic said:

Participant (6): *“I’ve been in the business for [many] years and we’ve been the good guys for [many] years* (laughs). *Well, I was at [big events], no matter. In many places they were fighting each other or whatever, but one thing was clear: we are the rescue service, we are the good ones and we help and we are neutral. We are not in this game. And that was the first time for me that we weren’t neutral. We were a party. Well, we were combatants [combating on the authorities’ side], too, to put it in this nasty jargon. And that’s something that was a really new experience for me, as someone who has been working for many, many years now.”*

Some participants of the emergency service group yet expressed the suspicion that the hunger strikers were influenced by a group of “ringleaders” and supporters who did not participate in the hunger strike themselves. Some suspected that the entire hunger strike was not primarily based on the strikers’ own initiative, but was “staged” by third parties to enforce their political interests.

#### 4.2.3 Medical consultants for the authorities

[1] Assignment description and role perception

Participants of the group of medical consultants were two physicians commissioned by the authorities to assess the medical situation. They were supposed to inform the authorities if a forced eviction of the camp was necessary. They saw their tasks in making recommendations and protecting the health and the lives of the hunger strikers. One medical expert, a physician working for the municipality described their role as follows:

Participant (9): *“We write medical reports […], so it is part of the job to get an impression of a person’s condition within a short time […] So, we then say: Well, if the camp continues to exist under these conditions, if people continue to starve and not drink, then these dangers can be expected (…) around that time. And then the district administration department decides whether to allow the camp to continue under these conditions or not.”*

The other medical consultant was a physician in a double function: as a representative of a physicians’ association to participate in the provision of health care and as medical consultant for the authorities.

[2] Medical tasks and challenges

One participant in this group stated that he/she had not taken any specific medical measures him-/herself, since his/her task was not to provide medical care for the strikers, but to provide medical expertise to the authorities. The other reported his/her provision of basic emergency measures during the first days of the hunger strike before providing expertise. Both participants assessed the situation as more critical than the participants of the other groups. They concluded that an acute serious danger to the strikers’ lives was very likely. One of them stated that he/she did not have the opportunity to directly examine the hunger strikers, as he/she was not allowed to enter the camp. He/she made his/her assessment based on statements from other health care professionals. One of them said:

Participant (8): “*So I saw the health situation and I mean, on some days five or six people were transported away in a somnolent to comatose state*. […] *I also said, I can’t rule out that somebody could have an epileptic seizure, someone could aspirate, someone could die.”*

Based on these evaluations they recommended the forced eviction of the camp to the authorities. They also mentioned fear of otherwise being held personally responsible in case of the death of a striker.

[3] Interaction with the hunger strikers

One medical consultant reported that he/she did not have direct contact with the hunger strikers:

Participant (9): “*I wanted to get an impression of the condition of the [hunger strikers], but the organizers did not agree that a representative of the municipality […]- that’s how they saw me, not as a doctor, but as a representative of the municipality […]—should enter the refugee camp. So, I was not allowed to examine a refugee.*

The second participant reported that he/she had access to the camp in the first phase of the hunger strike, when he/she was still involved in emergency health-care provision. Having revealed him-/herself as a medical consultant commissioned by the municipality, he/she was restricted from entering the camp by the “organizers” of the protest.

In accordance with the participants from the second group he/she stated that the medical emergency measures were always accepted by the hunger strikers. He/she described the contact with the hunger strikers as short and focused on the acute medical conditions without in-depth conversations about their will or values. Treatment decisions had to be made within a very short time.

[4] Role conflicts

One participant in the medical consultant group did not report role conflicts. It was obvious to him/her that he/she had a clear assignment, namely to provide the municipality with a medical expert opinion on the gravity of the situation.

The other participant stated tension arose due to his/her double function as a health care provider and a medical consultant. In his/her function as a medical expert commissioned by the authorities, he/she was not perceived as neutral by the “organizers” of the hunger strike, but rather as an “opponent”.

#### 4.2.4 Interactions between the three groups of medical professionals

The participants in the voluntary physician group described a good collaboration with the public emergency services, and from the perspective of participants in that group, the collaboration with voluntary physicians was also perceived as positive, smooth and uncomplicated. Conflicts seemingly arose among one of the voluntary doctors and one of the medical consultants with regard to the overall medical assessment. While the voluntary physician had the impression that the situation was under control, the medical consultant stated that the lives and health of the strikers were in imminent danger, recommending forced eviction. The voluntary physician expressed his/her suspicion that the responsible authorities were dramatizing the situation putting pressure on the medical consultant.

One of the medical consultants expressed the impression that those responsible in politics and administration were afraid of being held responsible in case one of the strikers died. For one of the voluntary physicians, his/her opposition to the forced eviction of the hunger strikers had legalconsequences but the charges were finally rejected as groundless.

## 5. Discussion

Our study provides insights into the lived experiences of health-care professionals in a public collective hunger strike of asylum seekers. To our knowledge, it is the first qualitative study in the German-speaking context that addresses health-care delivery during hunger striking and one of the first empirical studies of challenges of professional health-care provision during a public collective hunger strike. We described the perspectives of participants of different groups of doctors and health personnel on site with a focus on their professional role perceptions.

The hunger strike that underlies this study was perceived as a complex situation with different actors placing colliding expectations on the medical personnel. As recent publications on health care in hunger strikes in non-custodial settings suggested (Vanobberghen et al., 2019, 2020; Verein demokratischer Ärztinnen und Ärzte, 2014), the experience of health-care providers in a public collective hunger strike differ from those who provide health care in hunger strikes in institutional settings (Gulati et al., 2018).

While in the health care in prison and detention centres the assigned doctors mostly work for the prison or the authorities, in this public collective hunger strike different groups of health care providers were involved. They described their assignments with different attitudes towards the justification for an ongoing strike, they have taken on different roles (health-care delivery, emergency medicine and health monitoring), with differing obligations towards the conflicting parties, while they all shared the goal to protect and promote the strikers’ health and well-being. Participants of these three groups (voluntary doctors, public emergency services, medical consultants) reported various challenges and upheld different positions in the conflict. Almost all participants perceived role conflicts, with the exception of one of the participants from the medical consultant-group.

The focus of most papers addressing hunger striking in detention is the decision whether to respect the hunger strikers’ autonomous decision *or* to take medical measures (such as forced administration of nutrition and hydration) against their will to prevent physical harm or even death (Crosby et al., 2007; Fayeulle et al., 2010; Gulati et al., 2018; Kenny et al., 2004; Reyes, 2007). In contrast to this, in our study most participants reported no considerations of forced medical measures as the hunger strikers always agreed to the proposed medical treatment (e.g., intravenous infusions with liquids or hospital admissions) except in the case of the forced eviction based on the decision of the authorities’ crisis committee. For most participants, the conflict culminated in the question whether a forced eviction of the strikers’ camp was justified because of the health risks for the strikers if collective striking continued. A forced termination of the strike cannot be regarded as a medical but rather a regulatory measure. The evaluation of the overall medical situation before the event ranged from “never out of control”, as perceived by the majority of the interviewed voluntary doctors and some members of the public emergency service, to a “critical situation” that demanded action as stated by one of the medical consultants. This different assessment of the situation could be explained by different levels of information about the state of the strikers’ health. Furthermore, the differently perceived assignments and obligations of the groups of health-care professionals identified may also have led to the varying evaluations of the same situation. Although participants of all groups stated that they saw it as their primary task to make sure that nobody got harmed, those closer to the strikers seemed to have based their judgement on individual assessments, while those closer to the authorities seemed to rely on a population-and-health-risk-based judgement. Furthermore, a concern to be held publicly accountable for the serious harm or death of strikers might have led to a more critical assessment of the situation and subsequently to a recommendation for a forced eviction. Besides, in most hunger strikes the strikers opted for total fasting with continued uptake of liquids (Başoğlu et al.,^2006^; Eichelberger et al., 2014; Oguz & Miles, 2005; Silove et al., 1996; WMA, 2006), which can last up to several weeks with a manageable risk to the strikers’ health. In the case we examined, the hunger strikers went on dry fasting, which can lead to death within a few days. This could explain the high pressure on the medical personnel since the time for negotiations was very limited. Another stress factor could have been that the hunger strike took place in public and under permanent observation of bystanders and the media.

Establishing mutual trust is regarded as a crucial requirement for a working physician-patient relationship in general (Hoff & Collinson, 2017; Rasiah et al., 2020) and is even more important in the particular case of health care for hunger strikers in detention (Gulati et al., 2018; Irmak, 2015; Reyes et al., 2013; WMA, 2006, 2017). It is regarded as within a doctor’s responsibility to build trust in order to assess a hunger striker’s health status and will concerning possible medical interventions in light of his/her decision-making capacity (García-Guerrero, 2013; Gulati et al., 2018; Kenny et al., 2004; Silove et al., 1996; Wei & Brendel, 2010; WMA, 2017). In the context of prison hunger strikes, problems with building a trusting relationship are known when medical personnel is perceived by the hunger strikers as part of “the system” or the authorities (Karni, 2015). Similar experiences are described in our study by the members of the public emergency service and the medical consultants, and the one doctor who switched groups. The level of trust differed significantlyamong the groups of participants identified and influenced the quality of the relationship with the hunger strikers. As the group of the voluntary physicians was perceived as the only group that was totally independentof authorities, it was seen as impartial and hence enjoyied a higher degree of trust from early on. They were given direct access to the camp of the hunger strikers and their medical recommendations were taken seriously. The group of the public emergency services and the medical consultants were not perceived as impartial actors but rather as representatives of the authorities, which resulted in mistrust. This made it almost impossible for these participants to establish a physician-patient relationship that lived up to the professional role expectations for doctors who deliver health care in hunger strikes such as outlined in the WMA Declaration of Malta (WMA, 2017).

These requirements include the expectation that physicians should provide information on the potential medical implications of hunger striking in order to realize the strikers’ right to informed refusal (Gétaz et al., 2012; Gulati et al., 2018; Oguz & Miles, 2005; WMA, 2017). Within the camp, the possibility of confidential individual conversations with the hunger strikers in order to provide information, perform thorough anamnesis and discuss wishes regarding potential medical interventions was scarce even for the trusted doctors.

Participants reported that hunger strikers asked them for symptomatic measures like intravenous infusions or painkillers in order to reduce symptoms during the hunger strike. These measures—if they help the strikers to continue striking—could be seen not only as symptom relief but as enhancement of the ability for suffering during the strike. This is in line with the observation that strikers returned and resumed striking after being treated in a hospital. Thus, neither the (symptomatic) medical intervention nor the behaviour of the hunger strikers were aimed at recovery (Parsons, 1951), which could have easily been achieved by the resumption of fluid and food intake. Several of the participants expressed their unease about participating in the enhancement of the strikers’ ability to continue fasting. In their role as health-care professionals the participants felt committed towards the best interests and the well-being of their patients. But to prolong such a harmful state of hunger strike by providing medication was not perceived as consistent with this principle. However, the situation got even more complex when a voluntary doctor was asked for by and provided water to one of the strikers. In that situation the voluntary doctor perceived conflicting commitments towards the interests of the individual opposed to the interests of the collective of the hunger strikers, including the risk of losing the collective’s trust. The doctor decided to accept the demand of the collective. This illustrates how the hunger strikers’ common goals, such as risking life and health in order to achieve a certain goal, can distort decisions on health care for individual hunger strikers.

Several participants stated that they had the suspicion that the hunger strikers (or at least some of them) were not fully aware of the medical implications of their hunger strikes and that some of them were pressured by their peers or by supporters. Such a possibility has also been described in collective hunger strikes in prison (Irmak, 2015; Jacobs, 2012; Oguz & Miles, 2005; WMA, 2006). In this case, such a perception could have also been influenced by a stereotypic view of asylum seekers and migrants as weak and less competent (Kotzur et al., 2019) and not able to organize and carry out such a resolute protest action independently. How the hunger strikers themselves experienced the hunger strike and health care could be a subject for further research to complement, expand or challenge our findings.

The actual participation in the forced eviction of the strike was perceived as burdensome to the emergency service providers, which could be described as a form of moral constraint-distress (Fourie, 2015, 2017). Most participants described a feeling of “instrumentalization” by different parties. Members of the public emergency services described a dual loyalty situation. This is comparable to situations described in custodial settings: a feeling of obligation towards the hunger strikers, based on a trustful relationship in some cases and conflicting expectations by employers and the government authorities (Gulati et al., 2018). This conflict became especially salient in the question of forced eviction and its implementation. Gétaz et al. (2012, p. 4) suggest a shift from a bilateral “physician-patient-relationship” to a “triadic physician-patient-authority relationship” when describing the dynamics of medical decision-making in hunger strike. With regard to the hunger strike situation we examined, one might add a collective of hunger strikers (i.e., those who are fasting, in distinction to the group of the supporters who are not hunger striking) to the equation as a fourth group of stakeholders influencing the health care provision to the individual striker.

### 5.1 Limitations

This interview study was conducted several years after the event. Accordingly, the events in retrospective might have been remembered differently than they actually took place. The sample is pre-selected, because neither could all health-care professionals who had been involved in the strike be found nor were all of them willing to participate. Health-care professionals who provided medical care to the strikers in the hospitals were not approached for this study. It is possible that further moral decisions were being made there, such as whether the striker could return to the site after treatment (before the forced eviction). We consider the participants’ perceptions not as statements of neutral observers but stakeholders. Studies that aim to reconstruct a hunger strike from the perspectives of the strikers, authorities, the media or public observers might come to different descriptions of challenges. The conclusions we have drawn have to be tested in their applicability to similar situations, as each hunger strike is a unique situation with its own dynamics. More research is needed to allow for a judgement about the generalizability of the results.

Furthermore, the authors’ perspectives might have influenced this research. With regard to their own political opinions concerning the legitimacy of a public collective hunger strike of asylum seekers, all authors are in favour of granting the strikers a right to public protest if this is their free will. On the basis of various human rights and professional ethos of health-care personnel the authors also had a general sympathy towards the asylum seekers’ cause. The authors likewise felt sympathy towards the health-care personnel involved who were committed to the striker’s health and well-being as well as their right to freedom of assembly.

## 6. Conclusion

The hunger-strike situation we investigated took place in a public, politically charged context. As our findings suggest, the role of doctors in a public and collective hunger strike is far more complex than in a “regular” clinical setting, and even more complex than in the case of a hunger strike in detention. Hunger strikes are considered highly political acts and health care for hunger strikers can hardly be regarded as being detached from the political and social contexts (Bendtsen, 2019; Filc et al., 2014; Lederman & Lederman, 2017). This also applies to the public collective hunger strike that was described in our study. The problem of maintaining clinical independence when being confronted with conflicting loyalties and expectations, political interests and institutional pressure is described as a key challenge with regard to the physicians’ role in prison hunger strikes (Crosby et al., 2007; Dougherty et al., 2013; Gétaz et al., 2012; Gulati et al., 2018; WMA, 2017).

A public collective hunger strike of asylum seekers has commonalities with and differences from a hunger strike in detention, which suggests a shared but differentiated guidance for these situations. In agreement with Vanobberghen et al. (2019), we recommend more research on collective hunger strikes in different settings to form the basis for an expansion of the ethical guidelines for health care in hunger strikes, above all the *WMA Declaration of Malta*.
